# Competency development for a volunteer navigation program to support caregivers of people living with dementia: A modified e-Delphi method

**DOI:** 10.1177/14713012231216768

**Published:** 2023-11-17

**Authors:** Madison Huggins, Gloria Puurveen, Barb Pesut, Kathy Rush, Caitlin McArthur

**Affiliations:** Faculty of Health and Social Development, 97950 University of British Columbia Okanagan, Kelowna BC, Canada; Faculty of Health, 3688 Dalhousie University, Halifax, NS, Canada

**Keywords:** navigation, caregivers, dementia, volunteers, needs, competency development, e-Delphi

## Abstract

Caregivers of people living with dementia are pillars of the care community. Providing them with adequate support throughout their caregiving journey is essential to their quality of life and may also contribute to improving the care of people living with dementia. Nav-CARE (Navigation - Connecting, Advocating, Resourcing, Engaging) is a volunteer-led navigation program that provides support to older adults with life-limiting illnesses who are living in the community. However, Nav-CARE does not provide support directly to caregivers of people living with dementia. To adapt Nav-CARE to support caregivers, we needed to establish caregivers’ needs and the competencies volunteer navigators should be trained in to support caregivers to meet these needs. To do so, a modified e-Delphi method was utilized, which consisted of administering three sequential questionnaires to a panel of 35 individuals with expertise in a variety of dementia related domains. Through this, two final lists of 46 caregivers’ needs and 41 volunteer competencies were established to inform the development of volunteer navigator training curriculum. Findings suggest that trained volunteer navigators may be able to support caregivers of people living with dementia throughout the disease trajectory and can be used to inform the development of future dementia navigation programs.

## Background

Persons living with dementia require support from a multitude of interdisciplinary professionals, however, care-partners are pillars of the dementia care community. Care-partners are those who provide unpaid care and support to people living with dementia (Brodaty & Donkin, 2009). Care-partners can be family members, neighbours, and/or friends of people living with dementia. They spend an average of 6.5 years in their role and globally provide approximately 82 billion hours of care annually in the form of cooking, cleaning, assisting with maintaining personal hygiene, attending doctors’ appointments, making healthcare decisions, and performing a variety of other tasks (Cheng et al., 2020; Frias et al., 2020; Hovland, 2018; Jeste et al., 2021). This care is equivalent to the efforts of 40 million full-time workers (Cheng et al., 2020; Jeste et al., 2021). In Canada alone, estimates suggest there will be over 1 million care-partners by 2050 (Alzheimer’s Society of Alzheimer Society of Canada, 2022). When compared to care-partners of individuals with other chronic diseases, care-partners of people living with dementia have a higher level of unmet needs and burden, more family conflict, personal stress, and serious health problems, lower utilization rates of available services, and an overall shorter life expectancy (Bressan et al., 2020; Brown & Chen, 2008). However, even when care-partners adapt well to situations, think positively about their role and report feelings of personal growth, enjoyment, satisfaction, meaning and accomplishment (Laparidou et al., 2019; Ribeiro et al., 2019) it is important to support them to maintain their active involvement in caregiving.

Although research has focused on understanding care-partners' needs and the efficacy of existing programs in meeting those needs, care-partners emphasize that a greater understanding of the realities of caregiving and more adequate supports are needed (Alzheimer Society of Canada, 2022). Emphasis on the importance of care-partners has been influenced by relationship centered care (RCC), which is a central framework considered in dementia care (De Witt & Fortune, 2019). RCC emphasizes relationships and interactions among people living with dementia and those in their care communities. Such relationships are essential to understanding the experience of living with dementia, the foundation of therapeutic and healing activities, and the support required for those impacted by the condition (Allison et al., 2019; Nolan et al., 2004). Within RCC is the concept of personhood, which emphasizes the importance of respecting the needs, desires, and identity of care-partners and people living with dementia, while acknowledging the sociocultural influences that impact them (Hennelly & O’Shea, 2022; O’Connor et al., 2007). RCC has focused attention on the importance of care-partners, on care in community settings, and on the spiritual, social, and relational ramifications of caring for someone with dementia (Penrod et al., 2007). As a result, a range of interventions to support care-partners have been developed. Common interventions include support groups, befriending, information sessions, exercise classes, and care coordination (Huggins et al., 2023). Interventions intend to address a variety of care-partner outcomes including burden of care provision, depression and anxiety, health and well-being, quality of life, knowledge and skills, social outcomes, and health care services utilization (Huggins et al., 2023). While there is variation in the efficacy of interventions, common factors contributing to their success include their ability to be tailored to the unique needs of care dyads as well as adapt to their changing needs along the disease trajectory (Huggins et al., 2023).

The use of navigation interventions in dementia care has been emerging over recent years (Amjad et al., 2017; Backhouse et al., 2017; Bernstein et al., 2020; Black et al., 2019; Zwingmann et al., 2019). Dementia navigation programs vary but generally utilize a non-clinical staff member to provide person-centered care and assist care-partners and people living with dementia to access existing services and supports within their communities, ultimately improving care integration (Giebel et al., 2023; Kokorelias et al., 2023). These programs have contributed to improvements in the behaviours of people living with dementia, decreased their healthcare utilization, and improved care-partner burden (Amjad et al., 2017; Backhouse et al., 2017; Bernstein et al., 2019; Bernstein et al., 2020; Black et al., 2019; Zwingmann et al., 2019). Many of these programs provide support directly to people living with dementia rather than care-partners and an evaluation of the impact of programs on care-partners is of secondary focus (Amjad et al., 2017; Backhouse et al., 2017; Bernstein et al., 2020; Black et al., 2019; Zwingmann et al., 2019). As such, less is known about the best approaches to providing navigation support directly to care-partners and the potential benefits of doing so. However, it has been suggested that shifting the focus of programs to care-partners may lead to a greater understanding of care-partners' needs and the importance of their role, as well as lead to the development of more adequate supports (McCabe et al., 2016).

An example of a navigation program that provides support directly to people living with life limiting illnesses that could be adapted to support care-partners is Nav-CARE (Navigation - Connecting, Advocating, Resourcing, Engaging). Nav-CARE is an evidence-based volunteer led program that supports individuals in the community living with declining health (Pesut et al., 2022). It utilizes trained, experienced, and mentored volunteer navigators to support clients in their homes or over the phone throughout their illness journey (Pesut et al., 2022). Volunteers are trained to address quality of life needs, advocate for clients and families, facilitate community connections, and promote active engagement (Pesut et al., 2020, 2022). Through consistent visits, relationship building takes place and clients can be supported with present and future planning and decision making, provided with a social safety net, and encouraged to engage with life (Pesut et al., 2020). Currently, Nav-CARE does not provide support to care-partners of persons living with dementia. However, if adapted using a relational approach, many components of the program may be beneficial to care-partners and persons living with dementia. The purpose of this study was to establish the foundation needed to adapt the Nav-CARE program for use within the context of dementia care. As such, this study focused on determining care-partners' needs that could be met through the support of a volunteer navigator, and the competencies volunteer navigators should be trained in to meet these needs. Findings from this study can contribute to conceptualizations of a volunteer navigator role and the use of navigation in the context of dementia care.

## Methods

Ethics approval was obtained through the University Behavioural Research Ethics Board at the University of British Columbia. All panelists provided written consent to participate in the study.

### Study design

The research approach used in this study was a modified e-Delphi (electronic). The Delphi method is defined as “a multi-staged survey which attempts ultimately to achieve consensus on an important issue” (Keeney et al., p. 3, 2011). In this study, this method was used to achieve consensus on care-partners' needs and competencies volunteer navigators must be trained in to meet these needs. The modification was a change in the way that the first questionnaire was developed. Typically, the first questionnaire is developed inductively based upon first round feedback (Keeney et al., 2011). However, in this study, it was developed based upon a rapid review of systematic reviews examining care-partners' needs (Huggins et al., 2023). With such a strong body of evidence currently in existence, this was a more rigorous way of beginning the consensus process. The initial survey was piloted by an Advisory Board consisting of four care-partners of people living with dementia to ensure appropriate language was used and care-partners' needs were accurately reflected.

### Sample and recruitment

Panelists were recruited through email using convenience sampling between July 2021 and October 2021. Individuals were identified through personal and professional networks of the research team and by reviewing global literature to identify experts publishing on topics relating to dementia caregiving. An invitation to participate was sent through email to 100 potential panelists who had expertise in dementia, volunteerism, volunteer navigation and/or had lived experience as a care-partner of a person living with dementia.

### Data collection and analysis

Following recruitment, three sequential questionnaires were administered to the expert panel through the secure online survey software, Qualtrics (Qualtrics, Provo, UT). Sequential questionnaire links were emailed directly to panelists who were given one month to respond to each questionnaire. A maximum of three reminder emails were sent to panelists over each one month period. After one month, if a response was still not received the panelist was considered lost to follow up. This process took place over a six-month period to allow for data analysis and survey development between the administration of questionnaires.

### First questionnaire

The goal of the first questionnaire was to establish consensus on care-partners' needs that could be met through this program. Panelists were instructed to contextualize these needs as those that have the potential to be met through assistance from a trained volunteer navigator and rate their importance. The list of needs statements was developed by conducting a comprehensive rapid review of systematic reviews of the needs of care-partners of persons living with dementia. This list of 43 items was categorized into six themes; (1) connecting through supportive relationships, (2) physical and emotional health, (3) practical help (e.g., yard work, cleaning), (4) finding and gaining access to care related resources, (4) information, and (5) healthcare support. Panelists were asked to rate the importance of each need statement on a Likert scale of 1–5 (“not at all important” to “extremely important”). Descriptive statistics were run on all statements and consensus was defined when 60% of respondents rated the statement’s importance as 3 or greater (Keeney et al., 2011). If a needs statement did not achieve consensus, it was removed from the list of needs that was provided in the second questionnaire.

For each of the six themes, panelists were asked to provide additional care-partner needs they believed were important but were not included in the questionnaire. All responses were quoted verbatim, placed in a Word document, and analyzed manually using content analysis (Keeney et al., 2011). Statements were reviewed to determine if they were similar or dissimilar and grouped together or separated accordingly (Keeney et al., 2011). Following this grouping, each statement was reviewed again to determine if and how similar statements could be collapsed together into succinct summary statements (Keeney et al., 2011). Focus was placed on ensuring the summary statements were as close to the original wording as possible. Once summary statements were created, each statement was organized into categories to reflect key aspects of the responses (Keeney et al., 2011). Thematic labels were established by reviewing the statements in each category collectively to determine the most appropriate label for the grouping.

### Second questionnaire

The second questionnaire focused on establishing the knowledge, skills, and abilities volunteer navigators need to be trained in to support care-partners in meeting their needs. To accomplish this, the thematically organized list of needs statements developed based upon responses to the first questionnaire were provided to panelists. Panelists were asked to inductively think of and provide suggestions related to the necessary knowledge, skills, and abilities in an essay response box. All statements were analyzed using content analysis following the same process used in the first questionnaire (Keeney et al., 2011). Thematic category labels and summary statements that were developed were used as the competency categories and competency statements presented in the third questionnaire.

### Third questionnaire

The third questionnaire focused on gaining consensus on the appropriateness of each competency statement. Panelists were provided with a thematically organized list of competency statements that was developed based upon responses to the second questionnaire. Panelists were asked to rank the appropriateness of each item on a Likert scale ranging from 1–5 (“not at all appropriate” to “extremely appropriate”). Descriptive statistics were run on all variables and consensus on the appropriateness of competencies was determined using the same process as the first questionnaire. Panelists were also asked to provide a list of resources at the community, provincial, and national level that they found to be beneficial to care-partners through previous experiences.

## Findings

35 individuals completed the consent process. Therefore, the expert panel consisted of 35 experts with diverse ages, areas of expertise, and years of experience. All panelists (*n* = 35) responded to the first and second questionnaires. Thirty panelists responded to the final questionnaire, which reflects a 16.6% attrition rate. The majority of respondents were female (*n* = 34). Importantly, 37.14% of respondents self-identified as care-partners of people living with dementia. Panelist demographics are provided in Table 1, Panelist Demographics.

**Table 1.** Panelist demographics.

	*N*	% Of respondents
Age		
25–34	2	5.71
35–44	4	11.43
45–64	17	48.57
>65	12	34.3
Main area of expertise		
Family and/or friend care-partner of a person living with dementia	13	37.14
Clinical work with dementia	9	25.71
Volunteer navigation	6	17.14
Dementia research	5	14.29
Volunteerism with dementia programs	2	5.71
Years of experience		
1–15 years	22	59.09
>15 years	13	40.91
Gender		
Male	1	2.86
Female	34	97.14

### First questionnaire: Care-partners’ needs

Panelists rated the importance of 43 care-partners' needs statements organized into six categories. All needs statements achieved 80%–100% consensus, exceeding the minimum 60% consensus threshold (see Table 2 for results). Therefore, all statements were included in the second questionnaire and final needs statement list. Modes ranged from 3–4 for all needs statements, which indicated that most panelists rated needs as either “moderately important” or “very important”. All needs statements had a range of 2, 3 or 4 indicating some variation in responses. The means of all needs statements ranged from 3.36–4.91 indicating that the importance of each need was consistently rated highly. The needs statement with the highest average importance rating was under category (6) Healthcare Support; “identifying providers that can help them [care-partners]”. Content analysis of qualitative responses resulted in the development of three additional needs statements, which are provided in Table 2: Consensus on Care-Partners' Needs.

**Table 2.** Consensus on care-partners’ needs.

Domain	Importance rating Percentage of respondents who rated item 3–5 on a 5-point scale	Mode	Missing data	Mean
1. Connecting through supportive relationships				
Develop/strengthen relationships with the following individuals/groups…				
Other care-partners	91%	4	0	3.97
The person living with dementia	97.14%	5	0	4.23
Their family members	88.24%	4	1	3.85
Their friends	78.78%	4	2	3.36
Their wider social circle (e.g., employer, children’s teacher, school counsellor, neighbor)	81.81%	4	2	3.36
Their healthcare providers	97.14%	5	0	4
*Additional needs statement developed from qualitative responses to questionnaire 1: It is important to have someone (e.g., a trained volunteer) assist care-partners in identifying and negotiating stigma associated with dementia caregiving				
2. Physical and emotional Health				
Assist care-partners in meeting the following personal physical and emotional health needs…				
Engage in enjoyable activities	80%	4	0	3.54
Listen to their experiences and concerns	97%	5	0	4.60
Help identify their physical health needs	94.28%	4	0	4
Help identify their emotional health needs	100%	5	0	4.46
Help identify resources to support their physical health needs	100%	5	0	4.47
Help identify resources to support their emotional health needs	100%	5	1	4.60
Help to stay and/or become physically active	97%	4	0	4.03
Provide support with experiencing grief	100%	5	0	4.54
Help to access resources to manage chronic health conditions (e.g., depression, anxiety, heart conditions)	100%	5	0	4.37
Encourage the communication of personal and emotional needs	100%	5	0	4.60
3. Practical Help				
Assist care-partners with finding help to meet the following personal needs…				
Making adaptations to care-partners' homes to accommodate the person living with dementia	100%	4	0	4.06
Doing home maintenance	97%	4	0	3.91
Cooking	94.28%	3	0	3.63
Cleaning	94.28%	3	0	3.80
Completing yard work	88.58%	3&4	0	3.63
Shopping and errands	97.15%	4	0	3.83
Completing paperwork and applications for support services	100%	5	0	4.37
4. Finding and gaining access to care related resources				
Assist care-partners with finding and accessing care-related resources…				
Programs that support their needs (e.g., health authority services, respite, support groups, financial assistance)	100%	5	0	4.63
Health assessments for the person living with dementia (e.g., cognition assessments if their condition seem to be worsening, occupational therapist assessments for the physical environment)	97%	5	0	4.51
Consistent and coordinated support from healthcare providers (e.g., from the dementia diagnosis onwards)	100%	5	0	4.63
After hour programs (e.g., someone to call for support, someone to come over to help with care outside of regular work hours)	100%	5	0	4.51
Tailored programs that are specific to the needs of the person living with dementia	97%	5	0	4.29
Comprehensive local support agencies (e.g., Alzheimer’s Society)	94.29%	4	0	4.20
A document outlining the necessary personal information about the person living with dementia that is required to advocate on their behalf in a variety of ways	97.14%	5	0	4.29
*Additional needs statement developed from responses to questionnaire 1: It is important to have someone (e.g., a trained volunteer) assist care-partners with findings and accessing respite services				
5. Information				
Assist care-partners with finding and accessing information about…				
Dementia (e.g., risk factors, symptoms, what to expect throughout the disease trajectory, treatment)	94%	5	1	4.29
Engaging in conversations about dementia (e.g., telling their social and professional networks about the dementia diagnosis)	100%	4	1	4.09
Strategies to manage the person living with dementia’s health (e.g., physical and emotional)	100%	5	1	4.38
Helping the person living with dementia have independence	100%	5	1	4.32
Supporting the person living with dementia with personal care tasks	100%	5	1	4.12
Making physical adaptations to person living with dementia’s living environment (e.g., door chimes, removing dangers that may increase their risk of injury)	97%	5	1	4.24
Financial resources and support (e.g., government funding, care-partners tax benefits, subsidized care)	100%	5	1	4.47
*Additional needs statement developed from responses to questionnaire 1: It is important to have someone (e.g., a trained volunteer) assist care-partners with accessing information about how to improve their skills with computers to increase their access to additional information				
6. Healthcare Support				
Assist care-partners in meeting their healthcare related needs (for themselves and the person living with dementia)…				
Talking to healthcare providers	100%	5	2	4.39
Identifying providers that can help them	100%	5	2	4.91
Booking and scheduling appointments	93%	4	2	4
Finding health region specific resources	97%	5	2	4.85
Informing healthcare providers about their specific circumstances and needs	97%	5	2	4.73
Filling out healthcare paperwork	93%	5	2	4.24

Based on importance ratings and qualitative responses, a final list of 46 needs statements was developed. The similarities among needs statements were assessed and statements were recategorized into six thematic groupings: (1) relationship needs, (2) personal, physical, and emotional health needs (of the person living with dementia and/or dyad), (3) care-partners’ various personal needs, (4) finding and accessing care-related resources, (5) accessing information, and (6) care-partners’ and/or people living with dementia’s various healthcare related needs. This recategorization and labeling provided a thematically organized summative list of care-partners' needs that was provided to respondents in the second questionnaire.

### Second questionnaire: Knowledge, skills, and abilities

The second questionnaire presented 46 needs (43 original needs and 3 additional needs from the first questionnaire) organized in six thematic groupings. Qualitative responses of the requisite knowledge skills and abilities for each need were reviewed and thematically organized. Content analysis of these responses resulted in the development of seven competency categories, and 41 sub-competencies. These competencies are provided in Table 3.

**Table 3.** Consensus on volunteer competencies.

Domain	Appropriateness rating	Mode	Mean	Missing data
1. Volunteer relational skills	*Percentage of respondents who rated item 3-5 on a 5- point scale*			
Communicate effectively with care-partners and people living with dementia	100%	5	4.90	0
Assess care-partners and people living with dementia’s physical, social and emotional needs	93.10%	4&5	4.07	0
Determine care-partners' preferred ways of communication	100%	5	4.52	0
Support care-partners while being open, empathetic, compassionate, and non-judgmental	100%	5	4.79	0
Develop positive conversations about aging and memory loss	96.55%	4	4.10	0
Understand ethical volunteer/client relationships including prioritizing confidentiality	100%	5	4.79	0
2. Supporting Care-Partners' well-being				
Determine how to support care-partners in establishing and maintaining social connections	97%	4	4.17	0
Identify creative ways to provide adequate support to care-partners	100%	4	4.17	1
Identify the “big picture” issues and be able to prioritize needs/tasks	97%	4	4.10	0
Identify common emotional experiences care-partners may be encountering and when to assist care-partners in accessing professional help	100%	4	4.30	0
Build capacity in supporting care-partners and people living with dementia with grief, vulnerability, depression and anxiety	93.33%	4	4.13	0
Assist care-partners in learning and engaging in self-advocacy	93.10%	5	4.10	1
Assist care-partners with utilizing technology (e.g., computer searches)	97%	4	3.97	0
Assist care-partners in communicating about grief and burnout	97%	4	4.23	0
Assist care-partners in self-assessing their personal health and well-being	100%	5	4.33	0
Assist care-partners in determining their willingness to accept support from different resources and their preferred way to engage with and/or access these resources (e.g., a care-partners may prefer online support, a care-partners may not want support from a specific organization)	93.33%	4&5	4.03	0
Identify respite options and ways to assist care-partners in accessing them	100%	4&5	4.30	0
3. Supporting the care dyad				
Determine what is meaningful to the care dyad	100%	4&5	4.30	0
Determine the best way to build a relationship with the care dyad (e.g., what kind of communication works best, how they like to interact with healthcare providers)	100%	4	4.37	0
Assess and assist care-partners in determining what relationships may need strengthening (e.g., with extended family members, with healthcare providers, between the care dyad)	97%	3	3.73	0
Assist care-partners in navigating conflict with the person living with dementia and/or family members	83.33%	5	3.87	0
Advocate to meet the dyad’s needs	100%	4	4.17	0
Assist care-partners in determining quality-of-life concerns for themselves and their person living with dementia	100%	4	4.37	0
4. Identifying and Assisting with accessing healthcare services				
Know care-partners and dementia support services available in the community and online (e.g., identifying beneficial resources at the community, provincial and national level)	100%	5	4.47	0
Identify how the healthcare system functions (especially regarding dementia related services) and assist care-partners in developing their understanding	97%	5	4.30	0
Identify services and assist care-partners in accessing them (e.g., completing paperwork and required forms, accessing financial resources and supports)	97%	5	4.33	0
Assist care-partners in overcoming barriers to accessing various services	100%	5	4.43	0
Assist care-partners with planning access to services they may need in the future	100%	4	4.23	0
Assist care-partners in tracking relevant healthcare information regarding the person living with dementia	93.1%	4&5	4.07	1
5. Knowledge of dementia				
Communicate information about dementia with care-partners and their social networks (e.g., help to explain what dementia is and/or what the disease trajectory entails to members of the care-partners' social network)	93.33%	4	3.97	0
Identify credible sources of information related to dementia and various associated health conditions	100%	5	4.37	0
Assist care-partners in accessing information about dementia	100%	4	4.30	0
Assist care-partners in contacting providers who can inform them about dementia	93.33%	4	3.93	0
Assist caregivers to identify strategies and support for engaging in challenging conversations about dementia (e.g., advanced care planning, changing abilities)	100%	5	4.37	0
6. Knowledge of dementia stigma				
Identify personal biases related to dementia	97%	5	4.27	0
Assess how stigma is encountered by care-partners and people living with dementia	100%	4	4.40	0
Assist in the co-development of a plan to negotiate and respond to experiences related to stigma	96.55%	4	4.28	1
7. Support for volunteers				
Build capacity to reflect on your experiences	97%	5	4.40	0
Determine how to practice self-care and self-compassion while in a volunteer role	100%	5	4.73	0
Build capacity in setting boundaries between yourself and the care-partners	100%	5	4.79	1

All competency statement categories were intended to adequately reflect the range of qualitative responses. For example, a respondent highlighted the importance of volunteer relational skills by stating it would be important for volunteers to have “strategies to build trust [and] how to define boundaries explaining [the] role of the volunteer navigator”. The importance was also highlighted by a respondent who stated “In relation to areas such as grief and sadness, the volunteer navigator not only needs to know the resources in their local community but must understand proper communication strategies, how to actively listen and be able to recognize if the dementia care-partner requires professional help following a discussion (i.e., becoming triggered or at risk of harming themselves)”. Respondents noted that it is important to support care-partners' wellbeing by stating volunteers need to have the “ability to help care-partners determine what [their] needs are” and the “ability to discern when assistance may be useful and how to introduce ideas in a gentle, empowering way”. The importance of supporting the care dyad was highlighted in statements from respondents suggesting volunteers need to take a “collaborative approach, tailoring supports to individual situations”. The importance of volunteers having knowledge of dementia was highlighted as a respondent noted the need for volunteers to have an “awareness of [the] trajectory of dementia, symptoms, and challenges that may be encountered”. This importance was also highlighted by a respondent who noted that a volunteer “should have good working knowledge of dementia and the complications of the disease and the challenges care-partners encounter when working with dementia”. Understanding dementia stigma was highlighted by respondents who stated volunteers need to have an “awareness of the stigma encountered by people living with dementia and their care-partners” as well as be able to “codevelop a plan to frame/negotiate experiences related to stigma. A respondent also highlighted the importance of having support for volunteers; “a good support system is important for those who may take on these volunteer roles. This can be through their spiritual supports, family or friends but also should be provided by the agency in which they volunteer for”. To identify and assist with accessing healthcare services, respondents highlighted the need for volunteers to have “strong knowledge of the healthcare system within their areas, including the gap and services and what other programs are available to fill those gaps (if any)” and be able to “actually make these connections [with healthcare providers] and set up the services as care partners are so overwhelmed with day-to-day care that they don’t have time to make phone calls and investigate options”.

In many cases, panelists provided responses that discussed specific programs or aspects of programs they have encountered that were either ineffective or effective at supporting care-partners. These responses highlighted a range of topics such as online resources, support groups, researching resources, and respite. As a result of these responses, an open-ended question was added to the third questionnaire to gather additional information on existing programs and supports.

### Third questionnaire: Appropriateness of competencies

The third questionnaire presented panelists with a list of 41 competency statements grouped into the seven competency statement categories outlined previously and asked them to rate the appropriateness of the competencies. Based on responses to this questionnaire, all competency statements remained on the list as they were deemed appropriate with 83.33%–100% agreement ratings. Appropriateness ratings for competency statements had ranges of 1, 2, 3 or 4, which indicated variation among responses. Modes for all competency statements ranged from 3–5, which indicated that the appropriateness of statements were often highly rated with some variation. The means for all competency statements ranged from 3.73 – 4.9, which indicated consistently high appropriateness ratings. The needs statement with the highest average appropriateness rating was “communicate effectively with care-partners and people living with dementia” in the category of Volunteer Relational Skills (see Table 3). Responses to the additional qualitative question regarding the efficacy of existing programs were used to develop a resource list.

## Discussion

The purpose of this study was to establish consensus regarding care-partners' needs and the competencies volunteer navigators must be trained in to meet them. Through this modified eDelphi study, two distinct comprehensive lists were developed: (1) care-partners' needs and (2) volunteer navigator competencies. Collectively, these lists outline the needs of care-partners that should be addressed through a volunteer-led navigation program, and the competencies volunteer navigators must be trained in to support care-partners to meet those needs. This study established the needs of care-partners that a volunteer navigator could meet which relate to six domains: (1) relationship needs, (2) personal, physical, and emotional health needs (of the person living with dementia and/or dyad), (3) care-partners' various personal needs, (4) finding and accessing care-related resources, (5) accessing information, and (6) meeting healthcare related needs. It also established the competencies volunteer navigators should be trained in, which relate to seven domains: (1) volunteer relational skills, (2) supporting care-partners’ well-being, (3) supporting the care dyad, (4) identifying and assisting with accessing healthcare services, (5) knowledge of dementia (6) knowledge of dementia stigma, and (7) support for volunteers. These needs and competencies align with dementia navigation literature, which highlights the need for programs to support care-partners, provide emotional support, coordinate with primary care providers, and tailor education programs and resources to care dyads’ unique needs (Amjad et al., 2017; Backhouse et al., 2017; Bernstein et al., 2019; Black et al., 2019; Zwingmann et al., 2019).

As such, this study has provided an important foundation to developing a volunteer-led dementia navigation program. However, there are several limitations to consider when evaluating these findings. The expert panel consisted of 35 individuals with diverse expertise relating to dementia. Despite the broad range of expertise, this was a small panel of experts. Additionally, the sample was primarily comprised of women and therefore, additional insights from a more gender diverse sample were not included. While some demographic data were collected, we did not collect data on ethnicity or social class. As such, we do not have an understanding of how these factors may have influenced perspectives. As the questionnaires were administered electronically, only individuals who had access to a computer and an understanding of how to use it were able to respond. While this allowed us to include the perspectives of experts across Canada, it also may have excluded valuable perspectives from those who do not have computer access. Finally, experts were provided with the questionnaires and given one month to respond. As they were not completing the questionnaires with a researcher present, they were unable to ask clarifying questions in real time. Therefore, confusion regarding the questions may have resulted in responses that do not adequately represent their perspectives. To mitigate this risk, a care-partner Advisory Board reviewed the wording of questionnaires prior to their administration and experts were made aware that they could contact the research team via email for clarification.

All competencies developed through this study are essential to the success of this volunteer-led dementia navigation intervention. For example, the competencies pertaining to volunteers’ relational skills and their ability to support a clients’ wellbeing, as well as their own, are foundational to the efficacy of a navigation program (Pesut et al., 2022). While all competencies require an understanding of the experiences of care dyads, four competencies - knowledge of dementia, knowledge of dementia stigma, supporting the care dyad, and identifying and assisting with accessing healthcare services - require additional consideration and a deep understanding of the nuances of the dementia journey that is beyond that of a typical volunteer. For example, volunteer roles that are task-related or require minimal contact between the client and the volunteer may not facilitate the type of relationship development envisioned in this navigational role. In the context of volunteer navigation with care-partners of persons living with dementia, the volunteer navigator will engage in consistent visits over a long period of time, get to know dyads personally, understand how dementia has impacted intimate aspects of their lives, and ultimately develop a meaningful relationship. Nuances of the experiences of dementia may impact the ways in which volunteers, care-partners, and people living with dementia interact with one another and the services in their communities. As such, consideration of these factors is essential to effectively train volunteer navigators, support care-partners, and improve the care of people living with dementia.

### Developing knowledge of dementia & dementia stigma

Panelists in this study identified the need to access relevant information about dementia and dementia stigma. As such, the competencies, knowledge of dementia and dementia stigma, generally refer to volunteer navigators supporting care-partners to ensure they have knowledge of multiple aspects of the disease and are able to communicate this with their wider social communities. The need for improved knowledge among care-partners and their wider social communities is well-established in the dementia literature (Ebert et al., 2020). Care-partners often highlight their lack of understanding of dementia and desire for more information on caregiving related topics (Peterson et al., 2016). It is important to ensure care-partners have adequate knowledge of the disease as this can develop care-partners’ confidence, positively impact their experiences of burden, anxiety, and depression, improve the care of people living with dementia, and delay placement in long-term care homes (Peterson et al., 2016; Tan et al., 2021; Teichmann et al., 2022). However, there are several complex barriers to improving care-partners' knowledge (Peterson et al., 2016). For example, information from primary care providers, who are often the key point of contact, can be limited and few educational programs are widely available (Peterson et al., 2016). Further, there may be a lack of knowledge of dementia in lay persons who have not been impacted by the disease or engaged in dementia education (Kim et al., 2019). As such, volunteers can play a key role in supporting care-partners to engage in more successful interactions with health care providers, while subsequently addressing stigma.

Findings from this study suggest that volunteers can support care-partners to use their knowledge in interactions with healthcare providers. Care-partners have highlighted a lack of dementia knowledge among healthcare providers (Peterson et al., 2016), which can present in the form of negative attitudes, lack of skills, and poor detection and management of dementia (Lv et al., 2021). This has been attributed in part to the lack of dementia specific education across provider programs (Keane et al., 2020). Mistrust in providers is significant as it could perpetuate negative experiences of caregiving such as increases in care-partner stress regarding the quality of care the person living with dementia is receiving, and a lack of opportunities for care-partners to learn more about the disease through communication with providers (Peterson et al., 2016). Improving the knowledge about dementia among healthcare providers is a complex and challenging task. However, volunteers can support care-partners to access information on dementia, which will enable them to plan for visits with healthcare providers by identifying the relevant questions they need to ask, symptoms they want to discuss, and programs about which they may want more information.

Volunteer navigators are uniquely positioned to play an instrumental role in improving knowledge of dementia among care-partners and their communities. Providing training to volunteer navigators regarding specific information about dementia, such as different diagnoses, disease trajectories, and common behaviours can contribute to informed volunteer-care-partner discussions. With this knowledge, care-partners will be able to engage in conversations with their wider social communities about the realities of dementia and share information that may not be well understood within communities. Providing information about dementia directly to care-partners may also be beneficial. However, support from the volunteer navigator, who has received specific education and conducted independent research using reputable sources, may contribute to reducing care-partners' burden. Further, volunteer navigators can improve their personal knowledge of dementia by interacting and establishing relationships with care-partners and people living with dementia (Harris & Caporella, 2014; Kim et al., 2019). Through volunteer care-partner engagement and the development of relationships, knowledge can go beyond a basic understanding of what constitutes dementia and instead include the nuances of what a dementia diagnosis means personally for those who are impacted (Ebert et al., 2020). This personhood-based knowledge is essential to improving comfort with dementia and reducing the perpetuation of stigma (Ebert et al., 2020) and may indirectly improve relationships between people living with dementia and their care-partners.

Lack of knowledge among providers and lay persons may contribute to developing and perpetuating stigma (Kim et al., 2019). Dementia stigma is important to address as it has tangible impacts on the health of care-partners and people living with dementia (Kim et al., 2019). For example, relationships between care-partners and their social networks can be negatively impacted by a lack of deep understanding of the caregiving experience (Ashworth, 2020). Further, experiences of stigma can contribute to increases in physical and psychological symptoms of dementia (Ashworth, 2020), and care-partners can experience shame and guilt, anticipatory grief, social exclusion, increased burden and develop negative self-perceptions (Ashworth, 2020; Su & Chang, 2020). As such, it is important that care-partners and people within their communities have knowledge of dementia to establish environments where social inclusion and positive conversations can take place, care-partners can be supported, and stigma and misconceptions can be addressed (Kim et el., 2019). Informed discussions about dementia, which may occur as a result of increased knowledge in volunteer navigators and care-partners, could reduce the spread of misinformation, the perpetuation of stigma and negative interactions with healthcare providers.

### Supporting the care dyad

Panelists in this study highlighted that care-partners need support with meeting their own personal, physical, and emotional health needs, those of the person living with dementia, and the relationship needs of the dyad. As such, supporting the dyad is a competency: one that requires volunteers to consider the best ways to engage with the dyad while honouring the needs and personhood of care-partners and people living with dementia. Supporting the dyad is essential as the wellbeing of the care-partner is intimately connected to the disease progression, the wellbeing of the person living with dementia, and the intricacies of their relationships (Stockwell-Smith et al., 2018). Therefore, these areas of support require consideration of both individual and relational needs and emphasize the importance of utilizing a RCC approach (Ryan et al., 2008). For example, while supporting care-partners, volunteers may need to consider the intricacies of verbal and nonverbal interactions between themselves and dyads, as well as the desires of the persons living with dementia.

Volunteers are in a unique position to support the care dyad as their role requires them to know the care-partner and person living with dementia on a deep and personal level. Personal knowledge about the care-partner and person living with dementia is needed to truly understand their values, complexities that influence decision making, and the best ways to offer support as their wellbeing is deeply connected to one another’s (Kim & Song, 2021; Stockwell-Smith et al., 2018). The volunteer navigator role provides the time and space to obtain this level of understanding and develop this relationship. Discussions between volunteers and care-partners that are able to take place as a result of this relationship may influence how care-partners make decisions, as well as the supports they access to address their own well-being throughout the caregiving journey. Supporting care-partners with decision making about the care of the person living with dementia may be a key role for volunteer navigators. In making these decisions, the care-partner may need to consider past conversations with the person living with dementia about their quality of life goals, and how they align with their own, as well as their caregiving capacity (Tranvåg et al., 2015; Waligora et al., 2019). If these conversations did not take place, care-partners may be in a position in which they are required to make decisions based on what they believe the person living with dementia would want. In some cases, the values of the person living with dementia and care-partners' own needs may be conflicting (Miller et al., 2018). Making these decisions can be extraordinarily challenging for care-partners and contribute to emotional distress (Fowler et al., 2013). Therefore, if volunteer navigators provide support to the care-partner early on in their journey, they may be able to encourage conversations between the dyad about quality-of-life goals in advance of acute and future events (Orsulic-Jeras et al., 2016). When providing support further along in the caregiving journey, volunteers may be able to provide emotional support to care-partners as they experience distress and grapple with these decisions (Fowler et al., 2013). As such, volunteers may be able to support care-partners in recognizing the importance of their own needs, as well as coping with grief and existential loss throughout the journey.

### Identifying and assisting with accessing healthcare services

Identifying healthcare services and assisting care-partners to access them is a competency that requires volunteers to provide meaningful navigational support. Panelists in this study identified the importance of volunteer support to find and access resources that can help care-partners meet their own personal, emotional and physical health needs as well as those of the persons living with dementia. There are many resources that care-partners may need support to access such as education programs, counselling, support groups, physical activity classes and respite (Whitlatch & Orsulic-Jeras, 2018). As such, it is important to train volunteers to navigate in these areas. For example, volunteer navigators need to be knowledgeable of the resources in their community and know how to encourage care-partners to engage with them.

Respite is an example of a healthcare service that volunteers can support care-partners to identify and access. Panelists in this study expressed that one of the most important services to support care-partners in their journey is respite. Respite can contribute to decreasing negative outcomes associated with the caregiving role such as burden and stress (Vandepitte et al., 2016). However, respite services are unavailable or inadequate in some communities (Bieber et al., 2019). There are several common barriers to accessing existing respite, which contributes to care-partners’ underutilization of these services (Bieber et al., 2019; Vandepitte et al., 2016). These barriers include care-partners’ concerns for the comfort of the person living with dementia when placed in long-term care or receiving home supports, guilt regarding leaving the person living with dementia, as well as the inadaptability, high cost, and lack of awareness of services (Gresham et al., 2018; Harkin et al., 2020; Vandepitte et al., 2016). Further, care-partners may be unaware of or unwilling to acknowledge their own needs and ask for help and lack support to help them do so (Zwingmann et al., 2019). Although some of these barriers are a result of systemic issues, volunteer navigators can address some barriers by providing an informed outsider perspective. With this perspective, volunteer navigators can encourage care-partners to recognize the potential benefit of respite and other services, as well as highlight the value in addressing their own needs. Volunteers can also support care-partners with practical tasks such as identifying respite services in the community, understanding the paperwork process required to apply for services, and encouraging care-partners to utilize these resources. Although volunteer navigators do not visit directly with people living with dementia or provide respite services, they can serve as a form of mental respite for care-partners by providing consistent visits, being present, listening, supporting, and establishing trust, which may allow care-partners to focus on their own needs.

## Research impacts and implications

Findings from this study broaden the notion of what dementia navigation interventions can entail, who can provide it, and how volunteers can be trained to provide such services. Much of the existing dementia navigation research utilizes trained navigators or coordinators who are paid employees, supported by clinical teams, and/or are clinically trained themselves (Bernstein et al., 2020; Jennings et al., 2019; LaMantia et al., 2015; Merrilees et al., 2018). The use of trained volunteer navigators with diverse backgrounds is less common and, therefore, not well understood. From a practice perspective, findings from this study suggest that utilizing volunteers to provide navigational support in a variety of domains may be an effective way to meet care-partners' needs. Recognition of the need for additional support for care-partners is reflected in The World Health Organization (WHO) Dementia Action Plan, which highlights support for care-partners as a priority action area and brings this issue to global audiences (WHO, 2017). Expanded conceptualizations of navigation and subsequent increased reach of these programs may be an effective way to meet care-partners' needs, thus helping to meet important policy priorities such as those contained in the WHO action plan (World Health Organization, 2017). As such, findings can be used by researchers and healthcare and community organizations to assist in guiding the development of dementia navigation programs, or to enhance training curriculum for volunteers who work with care-partners and people living with dementia.

## Conclusion

As the older adult population continues to increase, so too will the reliance upon care-partners (Alzheimer Society of Canada, 2022). Although there are programs to support care-partners many of their needs remain unmet (Bressan et al., 2020; Brown & Chen, 2008; Zwingmann et al., 2019). Programs that utilize trained volunteer navigators may serve as an effective approach to addressing care-partners' needs and improving supports. As researchers continue to investigate ways to support care-partners, it is imperative that the needs most important to care-partners are considered in both service provision and evaluation. Navigation programs provide an opportunity to honor those needs by valuing the development of meaningful relationships, understanding the unique needs of care dyads, supporting them to address these needs and alleviating the challenges care-partners face in meeting them. In doing so, care-partners' experiences may be improved and the wellbeing and connectedness of communities strengthened. Further, increased use of navigation programs in dementia care can help to expand conceptualizations of the ways in which care-partners can be supported. It is imperative that we continue to develop innovative strategies to support the growing population of care-partners as they are a group who are often forgotten, yet essential to dementia care.

## References

[bibr1-14713012231216768] Allison T. Balbino R. Covinsky K. (2019). Caring community and relationship centred care on an end-stage dementia special care unit. Age and Ageing, 48(5), 727–734. 10.1093/ageing/afz030 31220199

[bibr2-14713012231216768] Alzheimer Society of Canada . (2022). Navigating the path forward for dementia in Canada/The landmark study/Path. https://alzheimer.ca/sites/default/files/documents/LandmarkStudy-1-Path-Forward-Alzheimer-Society-of-Canada-2022-wb.pdf

[bibr3-14713012231216768] Amjad H. Wong S. K. Roth D. L. Huang J. Willink A. Black B. S. Johnston D. Rabins P. V. Gitlin L. N. Lyketsos C. G. Samus Q. M. (2017). Health services utilization in older adults with dementia receiving care coordination: The MIND at home trial. Health Services Research, 53(1), 556–579. 10.1111/1475-6773.12647 28083879 PMC5785326

[bibr4-14713012231216768] Ashworth R. (2017). Perceptions of stigma among people affected by early- and late-onset Alzheimer’s disease. Journal of Health Psychology, 25(4), 490–510. 10.1177/1359105317720818 28810495

[bibr6-14713012231216768] Backhouse A. Ukoumunne O. C. Richards D. A. McCabe R. Watkins R. Dickens C. (2017). The effectiveness of community-based coordinating interventions in dementia care: A meta-analysis and subgroup analysis of intervention components. BMC Health Services Research, 17(1), 717. 10.1186/s12913-017-2677-2 29132353 PMC5683245

[bibr7-14713012231216768] Bernstein A. Harrison K. L. Dulaney S. Merrilees J. Bowhay A. Heunis J. Choi J. Feuer J. E. Clark A. M. Chiong W. Lee K. Braley T. L. Bonasera S. J. Ritchie C. S. Dohan D. Miller B. L. Possin K. L. (2019). The role of care navigators working with people with dementia and their caregivers. Journal of Alzheimer's Disease, 71(1), 45–55. 10.3233/JAD180957. PMID: 31322558.; PMCID: PMC7004209. PMC7004209 31322558

[bibr8-14713012231216768] Bernstein A. Merrilees J. Dulaney S. Harrison K. L. Chiong W. Ong P. Heunis J. Choi J. Walker R. Feuer J. E. Lee K. Dohan D. Bonasera S. J. Miller B. L. Possin K. L. (2020). Using care navigation to address caregiver burden in dementia: A qualitative case study analysis. Alzheimer's and Dementia, 6(1), Article e12010. 10.1002/trc2.12010. http://mc.manuscriptcentral.com/dementia PMC7201177 32377557

[bibr9-14713012231216768] Bieber A. Nguyen N. Meyer G. Stephan A. (2019). Influences on the access to and use of formal community care by people with dementia and their informal caregivers: A scoping review. BMC Health Services Research, 19(1), 88. 10.1186/s12913-018-3825-z 30709345 PMC6359781

[bibr10-14713012231216768] Black B. S. Johnston D. Leoutsakos J. Reuland M. Kelly J. Amjad H. Davis K. Willink A. Sloan D. Lyketsos C. Samus Q. M. (2019). Unmet needs in community-living persons with dementia are common, often non-medical and related to patient and caregiver characteristics. International Psychogeriatrics, 31(11), 1643–1654. 10.1017/s1041610218002296 30714564 PMC6679825

[bibr11-14713012231216768] Bressan V. Visintini C. Palese A. (2020). What do family caregivers of people with dementia need? A mixed‐method systematic review. Health and Social Care in the Community, 28(6), 1942–1960. 10.1111/hsc.13048 32542963

[bibr12-14713012231216768] Brodaty H. Donkin M. (2009). Family caregivers of people with dementia. Dialogues in Clinical Neuroscience, 11(2), 217–228. 10.31887/dcns.2009.11.2/hbrodaty 19585957 PMC3181916

[bibr13-14713012231216768] Brown J. Chen S. (2008). Help-seeking patterns of older spousal caregivers of older adults with dementia. Issues in Mental Health Nursing, 29(8), 839–852. 10.1080/01612840802182854 18649210

[bibr14-14713012231216768] Cheng S. Li K. Losada A. Zhang F. Au A. Thompson L. W. Gallagher-Thompson D. (2020). The effectiveness of nonpharmacological interventions for informal dementia caregivers: An updated systematic review and meta-analysis. Psychology and Aging, 35(1), 55–77. 10.1037/pag0000401 31985249

[bibr15-14713012231216768] De Witt L. Fortune D. (2019). Relationship-centered dementia care: Insights from a community-based culture change coalition. Dementia, 18(3), 1146–1165. 10.1177/1471301217708814 28523966

[bibr16-14713012231216768] Ebert A. R. Kulibert D. McFadden S. H. (2020). Effects of dementia knowledge and dementia fear on comfort with people having dementia: Implications for dementia friendly communities. Dementia, 19(8), 2542–2554. 10.1177/1471301219827708 30739490

[bibr17-14713012231216768] Fowler N. R. Hansen A. S. Barnato A. E. Garand L. (2013). Association between anticipatory grief and problem solving among family caregivers of persons with cognitive impairment. Journal of Aging and Health, 25(3), 493–509. 10.1177/0898264313477133 23428394 PMC3614338

[bibr18-14713012231216768] Frias C. E. Cabrera E. Zabalegui A. (2020). Informal caregivers’ roles in dementia: The impact on their quality of life. Life, 10(11), 251. 10.3390/life10110251 33113995 PMC7690694

[bibr19-14713012231216768] Giebel C. Reilly S. Gabbay M. Dickinson J. Tetlow H. Hogan H. Griffiths A. Cooper C. (2023). Dementia care navigation: A systematic review on different service types and their prevalence. International Journal of Geriatric Psychiatry, 38(8), Article e5977. 10.1002/gps.5977 37526320

[bibr20-14713012231216768] Gresham M. Heffernan M. Brodaty H. (2018). The going to stay at home program: Combining dementia caregiver training and residential respite care. International Psychogeriatrics, 30(11), 1697–1706. 10.1017/S1041610218000686 30019662

[bibr21-14713012231216768] Harkin D. J. O’Connor C. Birch M. R. Poulos C. J. (2020). Perspectives of Australian family carers of people with dementia on the 'cottage' model of respite: Compared to traditional models of residential respite provided in aged care facilities. Health and Social Care in the Community. 28(3), 850–861. 10.1111/hsc.12916. Epub 2019 Dec 20. PMID: 31863540; PMCID: PMC7Article 187172. 31863540 PMC7187172

[bibr22-14713012231216768] Harris P. B. Caporella C. A. (2014). An intergenerational choir formed to lessen Alzheimer's disease stigma in college students and decrease the social isolation of people with Alzheimer's disease and their family members: A pilot study. American Journal of Alzheimer's Disease and Other Dementias, 29(3), 270–281. 10.1177/1533317513517044 PMC10852642 24413542

[bibr23-14713012231216768] Hennelly N. O’Shea E. (2022). A multiple perspective view of personhood in dementia. Ageing and Society, 42(9), 2103–2121. 10.1017/S0144686X20002007

[bibr24-14713012231216768] Hovland C. (2018). Welcoming death: Exploring pre-death grief experiences of caregivers of older adults with dementia. Journal of Social Work in End-of-Life and Palliative Care, 14(4), 274–290. 10.1080/15524256.2018.1508538 30457443

[bibr25-14713012231216768] Huggins M. Pesut B. Puurveen G. (2023). Interventions for caregivers of older adults with dementia living in the community: A rapid review of reviews. Canadian Journal on Aging/La Revue canadienne du vieillissement, 42(3), 425–433. 10.1017/s0714980823000016 36799030

[bibr26-14713012231216768] Jennings L. A. Laffan A. M. Schlissel A. C. Colligan E. Tan Z. Wenger N. S. Reuben D. B. (2019). Health care utilization and cost outcomes of a comprehensive dementia care program for Medicare beneficiaries. JAMA Internal Medicine, 179(2), 161–166. 10.1001/jamainternmed.2018.5579 30575846 PMC6439653

[bibr27-14713012231216768] Jeste D. V. Mausbach B. Lee E. E. (2021). Caring for caregivers/care partners of persons with dementia. International Psychogeriatrics, 33(4), 307–310. 10.1017/S1041610221000557 33970060 PMC8752059

[bibr28-14713012231216768] Keane J. M. Franklin N. F. Vaughan B. (2020). Simulation to educate healthcare providers working within residential age care settings: A scoping review. Nurse Education Today, 85, 104228. 10.1016/j.nedt.2019.104228. http://mc.manuscriptcentral.com/dementia 31765870

[bibr29-14713012231216768] Keeney S. McKenna H. Hasson F. (2011). The Delphi technique in nursing and health research. John Wiley & Sons.

[bibr30-14713012231216768] Kim J. Song J. (2021). Personhood communication with persons with dementia: Concept analysis. Journal of Korean Gerontological Nursing, 23(4), 406–417. 10.17079/jkgn.2021.23.4.406

[bibr31-14713012231216768] Kim S. Werner P. Richardson A. Anstey K. J. (2019). Dementia stigma reduction (Deserve): Study protocol for a randomized controlled trial of an online intervention program to reduce dementia-related public stigma. Contemporary Clinical Trials Communications, 14, 100351. 10.1016/j.conctc.2019.100351 30997434 PMC6453665

[bibr32-14713012231216768] Kokorelias K. M. Shiers-Hanley J. E. Li Z. Hitzig S. L. (2023). A systematic review on navigation programs for persons living with dementia and their caregivers. The Gerontologist, 63(8), 1341–1350. 10.1093/geront/gnac054 35439813

[bibr33-14713012231216768] LaMantia M. A. Alder C. A. Callahan C. M. Gao S. French D. D. Austrom M. G. Boustany K. Livin L. Bynagari B. Boustani M. A. (2015). The aging brain care medical home: Preliminary data. Journal of the American Geriatrics Society, 63(6), 1209–1213. 10.1111/jgs.13447 26096394

[bibr34-14713012231216768] Laparidou D. Middlemass J. Karran T. Siriwardena A. N. (2019). Caregivers’ interactions with health care services – Mediator of stress or added strain? Experiences and perceptions of informal caregivers of people with dementia – a qualitative study. Dementia, 18(7-8), 2526–2542. 10.1177/1471301217751226 29385819

[bibr35-14713012231216768] Lv X. Zhao M. Li T. Yuan C. Zhang H. Pu C. Li Z. Zhang N. Yu X. Wang H. (2021). Effects of an enhanced training on primary care providers knowledge, attitudes, service and skills of dementia detection: A cluster randomized trial. Frontiers in Neurology, 12, 651826. 10.3389/fneur.2021.651826 34367045 PMC8342805

[bibr36-14713012231216768] McCabe M. You E. Tatangelo G. (2016). Hearing their voice: A systematic review of dementia family caregivers’ needs. The Gerontologist, 56(5), Article e70–e88. 10.1093/geront/gnw078 27102056

[bibr37-14713012231216768] Merrilees J. J. Bernstein A. Dulaney S. Heunis J. Walker R. Rah E. Choi J. Gawlas K. Carroll S. Ong P. Feuer J. Braley T. Clark A. M. Lee K. Chiong W. Bonasera S. J. Miller B. L. Possin K. L. (2018). The care ecosystem: Promoting self-efficacy among dementia family caregivers. Dementia, 19(6), 1955–1973. 10.1177/1471301218814121 30497302 PMC6541533

[bibr38-14713012231216768] Miller L. M. Lee C. S. Whitlatch C. J. Lyons K. S. (2018). Involvement of hospitalized persons with dementia in everyday decisions: A dyadic study. The Gerontologist, 58(4), 644–653. 10.1093/geront/gnw265 28379352 PMC6044333

[bibr39-14713012231216768] Nolan M. R. Davies S. Brown J. Keady J. Nolan J. (2004). Beyond ‘person-centred’ care: A new vision for gerontological nursing. Journal of Clinical Nursing, 13(s1), 45–53. 10.1111/j.1365-2702.2004.00926.x 15028039

[bibr40-14713012231216768] O’Connor D. Phinney A. Smith A. Small J. Purves B. Perry J. Drance E. Donnelly M. Chaudhury H. Beattie L. (2007). Personhood in dementia care: Developing a research agenda for broadening the vision. Dementia, 6(1), 121–142. 10.1177/1471301207075648

[bibr41-14713012231216768] Orsulic-Jeras S. Whitlatch C. J. Szabo S. M. Shelton E. G. Johnson J. (2016). The SHARE program for dementia: Implementation of an early-stage dyadic care-planning intervention. Dementia, 18(1), 360–379. 10.1177/1471301216673455 27738110

[bibr42-14713012231216768] Penrod J. Yu F. Kolanowski A. Fick M. Loeb S. J. Hupcey J. E. (2007). Reframing person centered nursing care for persons with dementia. Research and Theory for Nursing Practice, 21(1), 57–72. 10.1891/rtnpij-v21i1a007. PMID: 17378465; PMCID: PMC2 844333. 17378465 PMC2844333

[bibr43-14713012231216768] Pesut B. Duggleby W. Warner G. Ghosh S. Bruce P. Dunlop R. Puurveen G. (2022). Scaling out a palliative compassionate community innovation: Nav-CARE. Palliative Care and Social Practice, 16, 26323524221095102. 10.1177/26323524221095102 35592240 PMC9112317

[bibr44-14713012231216768] Pesut B. Duggleby W. Warner G. Kervin E. Bruce P. Antifeau E. Hooper B. (2020). Implementing volunteer-navigation for older persons with advanced chronic illness (NavCARE): A knowledge to action study. BMC Palliative Care, 19(1), 72. 10.1186/s12904-020-00578-1 32443979 PMC7245025

[bibr45-14713012231216768] Peterson K. Hahn H. Lee A. J. Madison C. A. Atri A. (2016). In the information age, do dementia caregivers get the information they need? Semi-structured interviews to determine informal caregivers’ education needs, barriers, and preferences. BMC Geriatrics, 16(1), 164. 10.1186/s12877-016-0338-7 27662829 PMC5035467

[bibr46-14713012231216768] Ribeiro O. Brandão D. Oliveira A. F. Teixeira L. Paúl C. (2019). Positive aspects of care in informal caregivers of community‐dwelling dementia patients. Journal of Psychiatric and Mental Health Nursing, 27(4), 330–341. 10.1111/jpm.12582 31811684

[bibr47-14713012231216768] Ryan T. Nolan M. Reid D. Enderby P. (2008). Using the senses framework to achieve relationship-centred dementia care services. Dementia, 7(1), 71–93. 10.1177/1471301207085368

[bibr48-14713012231216768] Stockwell‐Smith G. Moyle W. Kellett U. (2018). The impact of early‐stage dementia on community‐dwelling care recipient/carer dyads’ capacity to self‐manage. Journal of Clinical Nursing, 28(3-4), 629–640. 10.1111/jocn.14657 30182488

[bibr49-14713012231216768] Su J. Chang C. (2020). Association between family caregiver burden and affiliate stigma in the families of people with dementia. International Journal of Environmental Research and Public Health, 17(8), 2772. 10.3390/ijerph17082772 32316454 PMC7215659

[bibr50-14713012231216768] Tan H. Yuan Q. Devi F. Wang P. Ng L. Goveas R. Chong A. Subramaniam M. (2021). Dementia knowledge and its demographic correlates amongst informal dementia caregivers in Singapore. Aging & Mental Health, 25(5), 864–872. 10.1080/13607863.2020.1740914. PMID: 32228179. 32228179

[bibr51-14713012231216768] Teichmann B. Gkioka M. Kruse A. Tsolaki M. (2022). Informal caregivers' attitude toward dementia: The impact of dementia knowledge, confidence in dementia care, and the behavioral and psychological symptoms of the person with dementia. A cross-sectional study. Journal of Alzheimer's Disease: JAD, 88(3), 971–984. 10.3233/JAD-215731. PMID: 35723101; PMCID: PMC9484115. 35723101 PMC9484115

[bibr53-14713012231216768] Tranvåg O. Petersen K. A. Nåden D. (2015). Relational interactions preserving dignity experience: Perceptions of persons living with dementia. Nursing Ethics, 22(5), 577–593. 10.1177/0969733014549882 25319119

[bibr54-14713012231216768] Vandepitte S. Van Den Noortgate N. Putman K. Verhaeghe S. Faes K. Annemans L. (2016). Effectiveness of supporting informal caregivers of people with dementia: A systematic review of randomized and non-randomized controlled trials. Journal of Alzheimer's Disease: JAD, 52(3), 929–965. 10.3233/jad-151011 27079704

[bibr55-14713012231216768] Waligora K. Bahouth M. Han H. (2019). The self-care needs and behaviors of dementia informal caregivers: A systematic review. The Gerontologist, 59(5), Article e565–e583. 10.1093/geront/gny076 29931147

[bibr56-14713012231216768] Whitlatch C. J. Orsulic-Jeras S. (2018). Meeting the informational, educational, and psychosocial support needs of persons living with dementia and their family caregivers. The Gerontologist, 58(suppl_1), S58–S73. 10.1093/geront/gnx162 29361068

[bibr57-14713012231216768] World Health Organization . (2017). Global action plan on the public health response to dementia 2017–2025. Licence: CC BY-NC-SA 3.0 IGO.

[bibr58-14713012231216768] Zwingmann I. Hoffmann W. Michalowsky B. Dreier-Wolfgramm A. Hertel J. Wucherer D. Eichler T. Kilimann I. Thiel F. Teipel S. Thyrian J. R. (2019). Supporting family dementia caregivers: Testing the efficacy of dementia care management on multifaceted caregivers’ burden. Aging & Mental Health, 22(7), 889–896. 10.1080/13607863.2017.1399341 29156941

